# Effects of an Extract from Olive Fruits on the Physicochemical Properties, Lipid Oxidation and Volatile Compounds of Beef Patties

**DOI:** 10.3390/foods9121728

**Published:** 2020-11-24

**Authors:** Inmaculada Gómez, Celia García-Varona, María Curiel-Fernández, Miriam Ortega-Heras

**Affiliations:** Food Science and Technology Department, Faculty of Science, University of Burgos, Plaza Misael Bañuelos s/n, 09001 Burgos, Spain; igbastida@ubu.es (I.G.); cgv0029@alu.ubu.es (C.G.-V.); mcuriel@ubu.es (M.C.-F.)

**Keywords:** color, inhibition of oxidation, meat stability, modified atmosphere packaging, natural antioxidants, TBARS, volatile organic compounds

## Abstract

The aim of this work was to evaluate the effect of an olive extract (OE) on the physicochemical and microbiological characteristics, lipid oxidation and volatile compounds of beef patties stored both aerobically and under modified atmosphere packaging for 15 days at 4 °C. The antioxidant and antimicrobial effects of the OE were compared to those of sulfite. Four formulations were elaborated according to the antioxidant and dose used: without antioxidant, C; 300 mg potassium metabisulfite/kg product, S; 150 mg OE/kg product, OE1; and 250 mg of OE/kg product, OE2. The parameters analyzed were pH, water activity, color, lipid oxidation (TBARS and volatile organic compounds: hexanal, 2-pentyl-furan, 1-pentanol, 2,3-octanedione and nonanal, 1-octen-2-ol) and total viable counts. The OE delayed the loss of the bright red color of the patties and reduced the lipid oxidation in both types of packaging compared to the control patty. Sulfite was the most effective antioxidant for inhibition of the total viable counts. An OE could be used as a natural antioxidant to delay the lipid oxidation of meat without negatively affecting its physicochemical properties.

## 1. Introduction

Lipid and protein oxidation of meat is a key factor that most affects the quality and the acceptance of meat products and limits their shelf-life [1]. Meat has a bright red color, but the color turns brown over time due to protein oxidation, which is unattractive to the consumer. In this process, the oxymyoglobin is transformed into brown metamyoglobin through oxidation of the iron atom within its heme group [2]. Moreover, the taste and texture of the meat change as a result of the oxidation of fats, which also generate the compounds responsible for the unpleasant rancid odors. Certain operations, such as the grounding and mixing of meat [3], the addition of salt due to its prooxidant effect [4] and the presence of oxygen during the storage time, favor lipid oxidation. Likewise, the meat’s composition and fat content, together with the storage temperature, will also affect the oxidative process of the lipids.

Among the most used methods for meat conservation, the industry employs modified atmosphere packaging (MAP) to prevent protein oxidation and extend the shelf-life of fresh meat products. MAP is defined as the tailored mixture of atmospheric gases defined to replace the air surrounding the food in the package, whose composition differs from normal air [5]. A high amount of oxygen (between 60 and 80%) is added in the MAP package to keep the bright color of the meat; however, by contrast, high oxygen concentrations accelerate the growth of microorganisms that cause meat spoilage [6].

The shelf-life of meat products can be also extended by using antioxidants that delay lipid and protein oxidation. Sulfite is a widely used chemical antioxidant, which has also antimicrobial properties [7]. It is worth remarking that sulfite ingestion has been correlated with several adverse and toxic reactions, such as hypersensitivity, allergic diseases and vitamin deficiency, and may lead to dysbiotic events of the gut and oral microbiota [8]. In many countries, these additives are heavily regulated, and the legislation restricts their usage in meat products [8]. Currently the food industry needs to discover natural antioxidants that can replace chemical antioxidants [9]. For instance, the high polyphenol content of certain plant extracts can reduce oxidative reactions. Some of these extracts can be obtained from olive oil by-products, such as the leaves, pulp or juice [10]. Extracts obtained from olives contain significant amounts of water-soluble polyphenols, among which hydroxytyrosol stands out [11,12]. Hydroxytyrosol has several healthy properties, such as being antioxidant, anticancer, anticholesterol and antidiabetic, as well as protecting against oxidative stress and heart disease [13].

The antioxidant and antimicrobial effect of different products obtained from olive leaf extracts in meat products have been reported [14]. Thus, Hayes et al. [3] reported that an extract obtained from olive leaves inhibited lipid oxidation in beef patties packaged both in aerobic conditions and in a modified atmosphere, delaying the appearance of rancid odors. Moreover, Hayes et al. [15] and Hayes et al. [16] stated that an extract from olive leaves reduced lipid and oxymyoglobin oxidation in pork patties and pork sausages, respectively. Mondache et al. [17] studied the antioxidant effect of an active plastic film containing an olive leaf extract on fresh pork meat. In turn, Jimenez et al. [18] and Djenane et al. [19] reported the antioxidant effect of an olive leaf extract in frying oil and raw halal minced beef, respectively. Recently, Burri et al. [20] found that an olive polyphenol powder extracted from wastewater successfully inhibited the lipid oxidation of a processed meat model.

However, to our knowledge, no previous research has explored the potential of the extract obtained from olive fruit in meat products. Therefore, the influence of this product deserves research due to the current interest of the meat industry to replace chemical antioxidants by natural antioxidants. Likewise, the effect of an olive extract on the volatile organic compounds (VOCs) formed during the lipid oxidation of beef meat is worthy to explore. This issue is important as the different VOCs formed after lipid oxidation is conducive to rancid and unpleasant flavors and ultimately a reduced sensorial quality of meat products [21]. Furthermore, some of these VOCs, such as hexanal, are reliable parameters for monitoring the shelf-life of these products [22]. The aim of this study was to evaluate the effect of an olive extract (OE) on the physicochemical and microbiological characteristics, lipid oxidation and volatile compounds of beef patties stored both aerobically and under modified atmosphere packaging (MAP) for 15 days at 4 °C. The antioxidant and antimicrobial effects of the OE were compared to those of sulfite.

## 2. Materials and Methods

### 2.1. Raw Materials

Meat was obtained from beef skirt (*M. obliquus, transversus* and *rectus abdominis*) that was provided by a local supplier. The cattle selected came from a Spanish farm were 12 months old when slaughtered. They were fed with fodder grain based on wheat, barley, corn and soy. After collection, the meat was frozen until processed. A commercial olive extract (OE) (Hydroliv-Plus™) with a polyphenol content of 25% and hydroxytyrosol content of 15% (Biosearch Life, Granada, Spain) was used. Olive brine was used for the extraction; thus, this extract was from olive fruits. The OE was a homogeneous, yellowish, water-soluble powder. The OE levels assayed were 150 mg/kg of product (OE1) and 250 mg OE/Kg of product (OE2). The doses used were selected based on a previous study, which reported that a dose of 250 mg OE/Kg did not affect negatively the sensory parameters of beef patties [23] and delayed the lipid oxidation for 6 days of refrigerated storage (data not published). Other additives used were common salt (NaCl) (Carrefour S. A., Madrid, Spain), food-grade potato starch (Doscadesa 2000 S. L., Murcia, Spain) and potassium metabisulfite (Doscadesa 2000 S. L., Murcia, Spain).

### 2.2. Beef Patty Manufacture

Four formulations were elaborated according to the antioxidant and dose used: without antioxidant (C); 300 mg potassium metabisulfite/kg product (S); 150 mg OE/kg product (OE1); and 250 mg of OE/kg product (OE2).

Two different packaging methods were studied: MAP—modified atmosphere with a high-oxygen atmosphere (80% O_2_/20% CO_2_)—and an overwrap.

The meat was minced in a meat grinder (Cato, Talsabell S.A., Sabadell, Spain) to a particle size of 8 mm. The formulation employed is a modification of the one used in a previous study [7]. It corresponds to a traditional recipe for the elaboration of patties, although spices were not added. Control beef patties (C) were prepared by mixing 92.3% of the minced meat, 1.2% of the potato starch, 1.5% salt and 5% water. Sulfite samples (S) were similarly prepared by adding the corresponding quantity of potassium metabisulfite to obtain a final concentration of 300 mg/kg product. The beef patties with OE were formulated in the same way, using the corresponding amount of OE to obtain 150 mg OE/kg product (OE1) or 250 mg OE/kg product (OE2).

Ingredients were mixed manually. Patties of 120 g were formed with the help of a 10 cm in diameter and 1.5 cm-thick mold.

In the MAP packaging, the samples were packaged in trays of polyethylene/ethylene vinyl alcohol/polystyrene (Sanviplast, Barcelona, Spain) with a permeability to oxygen of 0.99 cm^3^/(m^2^ day atm). They were then sealed using a polyethylene terephthalate polyvinylidene chloride/polyethylene film with an oxygen permeability of 7 cm^3^/(m^2^ day atm) (Amcor Flexibles S.A., Burgos, Spain). The packing machine used was an Efabind efaman (Tobepal, Burgos, Spain). In the overwrap conditions, samples were packaged by sealing the film upon the tray.

Sampling of the refrigerated patties (4 °C) was conducted at days 0, 2, 5, 8, 12 and 15 of storage. Per formulation per packaging and per day of storage, two patties were used. Furthermore, two experiments or batches were carried out in different days. That means that 96 patties were used per experiment and thus 192 in total.

### 2.3. Proximate Analysis

The protein, moisture and total fat content were determined for each sample on Day 0 of the experiment by near-infrared spectroscopy using FoodScanLab equipment (FossAnalytical, Hillerød, Denmark).

### 2.4. pH and Water Activity

The pH was measured using a pH-meter Crison 2001 (Crison Instruments S.A., Barcelona, Spain) equipped with a glass probe for penetration. Two measurements were carried out at two randomly selected and non-overlapping points of the patties. The water activity (a_w_) was measured taking two grams from different points of the patty in order to homogenize the sample. AQUALAB CX2 (Decagon, Washington, EEUU) equipment was used for the determinations. The analyses were performed in duplicate.

### 2.5. Color

Instrumental measurement of the color of the patties was performed employing a Konica Minolta CM-2600d spectrophotometer (Konica Minolta Business Technologies Inc., Tokio, Japan). Color readings were taken at 5 randomly selected non overlapping points on the surface of each sample. Before the measurements, the patties were placed on a clean and white surface. Patty color was measured in the CIELAB space (International Commission on Illumination, 2004), with a standard illuminant D65 and an observer angle of 10°. The lightness (L*), redness (a*) and yellowness (b*) parameters were recorded. The total color difference parameter (ΔE*) between the two different products was calculated in CIELab units using the expression ΔE* = ((L_c_^*^-L_i_^*^)^2^ + (a_c_^*^−a_i_^*^)^2^ + (b_c_^*^−b_i_^*^)^2^)^1/2^. Two ΔE* values were calculated. Firstly, the color differences between the control patties (c) and the patties with the antioxidants, S, OE1 or OE2 (i) were calculated. Secondly, the color differences between patties at Day 0 (c) and the other days of storage (i) were calculated. In general, the human eye is able to discriminate two colors when ΔE* ≥ 2.3 [24].

### 2.6. Microbiological Analysis

Ten grams of meat, taken from different points of the patty in order to homogenize the sample, were aseptically placed into a stomacher bag. Then, the samples were homogenized with 90 mL of Ringer (Oxoid, England) in a laboratory blender (Stomacher^®^ 400, Seward, England) for 2 min at room temperature. For each sample, the appropriate serial decimal dilutions were prepared in Ringer solution. Total viable counts (TVC) were determined using pour plates of Plate Count Agar (PCA; Scharlau, Spain) after incubation at 30 °C for 48 h. Microbiological data were transformed into logarithms of the number of colony-forming units per gram (cfu/g). All measurements were carried out in duplicate.

### 2.7. Lipid Oxidation

Thiobarbituric acid reactive substances (TBARS) values were measured using the Tarladgis method [25]. Absorbance was measured at 538 nm in a spectrophotometer (model U-1900, Hitachi, Tokyo, Japan). The TBARS value was expressed as mg malonaldehyde (MDA)/kg patty. All measurements were carried out in duplicate.

### 2.8. Analysis of Volatile Organic Compounds (VOCs)

Volatile organic compounds (VOCs) were evaluated by a headspace solid phase dynamic extraction (HS-SPDE) coupled with a gas chromatography (Agilent Technologies HP 6890N, Agilent Technologies, S.L., Madrid, Spain) with a mass spectrometer (Agilent Technologies 5973, Agilent Technologies, S.L., Madrid, Spain) fully controlled by a CTC-CombiPAL autosampler (Bender and Hobein, Zurich, Switzerland), following the method proposed by Corcuera-Tecedor [26]. Three grams of each sample were introduced in a 10 mL glass vial (Chromacol Ltd. Herts. United Kingdom) and sealed using a metallic cap with a chlorobutyl/polytetrafluoroethylene seal (Chromacol Ltd. Herts). Extraction was conducted in an incubation station at 55 °C, using a previously conditioned SPDE-syringe with a non-polar PDMS/AC (90% polydimethylsiloxane and 10% activated carbon) needle (60 extraction strokes). After extraction, the VOCs were then desorbed from the fiber in the injection port at 250 °C, using helium as the carrier agent. Separation was conducted in a 007-WAX capillary column (Quadrex Corporation, New Haven, USA) (60 m length, 0.32 mm inside diameter and 1 mm film thickness). Compounds were identified by comparing their mass spectra with those found in the Wiley 7th and NIST 98 libraries and confirmed with their retention times. The results were expressed as the areas of each chromatographic peak’s (compounds) arbitrary units (AU). The VOCs analyses were carried out in triplicate, which means that 3 VOC measurements were carried out on each one of the two patties, elaborated per time, per formulation, per packaging and per experiment.

### 2.9. Statistical Analysis

The results are presented as the average ± standard deviation of the different replicates. To study the packaging effect, a Student’s t-test was performed. Two one-way ANOVAs were carried out to study the effect of “storage time” and of the “kind of antioxidant”. Differences between the means were analyzed by Tukey’s test. A two-way ANOVA with packaging and antioxidant was performed at each time of storage. The level of significance was set at *p* < 0.05 in all cases. Statistical analysis was carried out utilizing the package SPSS Statistics version 24 (IBM Corp., New York, NY, USA).

## 3. Results and Discussion

### 3.1. Proximate Composition

Table 1 shows the proximate composition of the beef patties. As expected, no differences were found among the four formulations (*p* > 0.05). That means that the small amount of antioxidants added does not affect the proximate composition of the patties. Moisture was around 70.9%, similar to the value reported in other studies [27,28]. The percentage of protein was around 19.5%, and this latter value was, in turn, higher than the value reported for conventional patties (16%) [18]. The fat content was around 7.7%, similar to the value reported in other studies [27].

### 3.2. pH and Water Activity

The effect of the addition of olive extracts on the pH and a_w_ of beef patties is shown in Table 2. All treatments had the same pH value at Day 0 (5.7). This pH value is similar to those reported by Hayes et al. [2], in beef patties with olive leaf extract, and Gómez et al. [27], in beef patties with grape seed extract.

Patties packaged in OW had pH values lower than those obtained in patties packaged in MAP at Day 2. During the rest of the days of refrigerated storage, the packaging did not affect the pH of the beef patties (*p* > 0.05).

There were significant differences among treatments (*p* < 0.05) for pH values from Day 8. The beef patties with sulfites kept their pH stable during the refrigerated storage, due to its antimicrobial and antifungal effect. However, the pH of the control patties and those with olive extract decreased from the 8th day of storage due to the lactic acid bacteria’s growth [29]. These results are in accordance with those found by Hayes et al. [3] in patties with and without olive leaf extract. Therefore, the addition of OE in the doses used in the present study does not affect the pH of the beef patties. The a_w_ values of the beef patties were not affected by packaging and type of antioxidant (*p* > 0.05) during the refrigerated store.

### 3.3. Color

Consumers’ acceptability is greatly affected by meat color. The combined effect of the addition of olive extract and the packaging method on the lightness (L*), redness (a*) and yellowness (b*) of beef patties refrigerated for 15 days is shown in Table 3.

Packaging affected the L* parameter (*p* < 0.05) from Day 8 and the lowest L* values were reached for the overwrap packages during the refrigerated storage. Thus, OW conditions led to a decrease in the L* parameter in beef patties. These results are in agreement with those obtained by Gómez and Lorenzo in foal meat [30]. Moreover, there was no observed effect for the antioxidant type used in the L* values (*p* > 0.05).

There were no significant differences among treatments (*p* > 0.05) for L* values during the refrigerated storage, except for the C-MAP treatment that had an increase in lightness until Day 12. Fernández-López et al. [31] also reported an increase in lightness during storage of ostrich steaks.

Redness is the most important parameter in red meat, since the bright red color is highly appreciated by the consumer. The packaging method significantly affected (*p*< 0.05) the a* parameter through all the days of the study. In MAP conditions, a* values were higher than those obtained in OW packaging up to 8 days of refrigerated storage. From the 12th day, the a* values in the MAP samples were lower than those obtained for the OW ones. These results agree with those found by several authors describing a faster decrease in a* when using MAP instead of overwrap conditions by the end of refrigerated storage [30,32].

The a* values showed significant differences (*p* < 0.05) among both packaging methods according to the antioxidant used. Thus, in the OW treatment, the OE did not allow to keep the redness of the meat since the samples with OE1 and OE2 showed similar values of the a* parameter than the control samples. However, if the addition of OE is combined with MAP, the redness preservation is achieved up to day 8 for the OE1 samples and 12 for OE2, respectively, since the a* values in the C treatments were lower than those obtained in treatments with OE added. There were no significant differences (*p* < 0.05) between the patties elaborated with the two doses studied for the a* parameter and, therefore, the lowest dose (150 mg/kg) would be enough in order to preserve the redness of the beef patties. These findings were in agreement with those reported earlier by Hayes et al. [3], who reported that the addition of an olive leaf extract improved the color stability in raw beef patties stored aerobically and in MAP for 12 days.

Moreover, all treatments showed a significant decrease (*p* < 0.001) in redness (around 66%) through the refrigeration storage time due to the pigment oxidation of meat products [2]. It should be noted that the C-OW treatment was the sample whose redness decreased faster. From Day 2, the sample had the same a* value observed for the rest of the refrigerated period.

The yellowness showed significant differences (*p* < 0.05) between the packaging conditions. The b* values were higher in the treatments packaged in the MAP conditions than those packaged in the OW ones. Thus, the MAP enhanced the b* stability in beef patties. Similar behavior was found by Gómez and Lorenzo [30] in foal meat.

The OE added at the doses studied protected the yellowness of the beef patties up to 5 days in the OW samples. However, in the MAP samples, no effect related to OE addition was noted.

Moreover, the yellowness was mainly affected by storage time (*p* < 0.001) and the b* values decreased during refrigerated storage of the beef patties.

As reflected in Table 4 the color differences found between the control patties and the patties with sulfites and the olive extracts were in general higher than 2.3. That means that consumers could detect the differences in color between the C-OW and C-MAP samples and their respective patties with antioxidants from Day 0 (with the exception of S-OW and OE1-OW) and over all the storage time.

The highest ΔE* values were found at Days 2 for the OW patties whereas in the MAP samples the highest values were reached at Day 8. An effect of the packaging or the antioxidant used was not found for this parameter, which means that consumers would not detect differences in color between the samples with sulfites or OE regardless of the packaging method used.

Table 5 shows the ΔE* values between the patties at Day 0 and the rest of the days. In this case, the ΔE* values were significantly higher than those found in Table 4, which means that the storage time clearly affect to the color of the patties due to the lipid oxidation and protein degradation. An effect of the storage time was found in all the patties except for C-OW and OE2-MAP. In the OE1-OW and OE2-OW patties, the color differences increased until Day 5 and then kept constant until Day 15. Meanwhile, the ΔE* values of the rest of the treatments increased until Day 8 and then kept constant. Therefore, the sulfite would protect the color more than the OE in OW conditions. Moreover, the MAP would enhance the color stability in beef patties in relation to the OW conditions, regardless of the use or not of antioxidants in the formulation.

### 3.4. Microbiological Analysis

Microbial spoilage usually reduces the shelf-life of raw meat. Table 6 reflects the effect of the addition of antioxidants and the packaging method on the total viable counts (TVC) of beef patties for refrigerated storage for 15 days. The initial TVC at Day 0 was 4.35 log10 cfu/g, a value similar to those obtained in raw beef patties at Day 0 [3]. The mincing process could contribute to the relatively high initial TVC in minced beef [3]. The addition of sulfite inhibited the growth of TVC (*p* < 0.05) from Day 8 of storage under both packaging conditions. This is related to the well-known antimicrobial effect of sulfite, one of the reasons for its use in the meat industry [7]. Conversely, the olive extract assayed did not show a bacteriostatic effect. Only a slight effect was observed at the higher doses used (250 mg/Kg) from Day 8. These results contrast with those reported by Hayes et al. [3], who found antimicrobial activity of an olive leaf extract in raw beef patties under aerobic and MAP storage. The different phenolic composition of the extracts studied, the doses added and the source, one from the olive fruit (the present study) and the other from the olive leaf [3], could explain why olive leaf extract was more effective against microorganisms than the olive extract.

Storage time had a significant effect on the TVCs of beef patties packaged in both the MAP and OW conditions, with an increase in TVC values (*p* < 0.001) observed over the 15 days of the refrigerated storage. The counts at which the meat becomes unacceptable by consumers, implying spoilage of the meat, is 7 log_10_ cfu/g [33]. Therefore, the shelf-life of beef patties without antioxidant (C) and with OE (150 or 250 mg OE/kg) stored under both MAP and OW conditions would be 8 days. The addition of sulfites would extend the shelf-life up to 12 or 15 days in MAP and aerobic storage, respectively. The microbiological results are correlated with the pH values as previously explained. The beef patties with sulfites had the highest pH values probably due to the lower proliferation of lactic bacteria and, therefore, a lower production of lactic acid.

Moreover, in spite of the presence of CO_2_, the MAP packaging did not inhibit microbial growth probably due to the growth of lactic bacteria, which are the predominant microorganisms in meat packaged in MAP [34].

### 3.5. Lipid Oxidation

Table 7 shows the effect of the addition of antioxidants and packaging method on lipid oxidation during 15 days at 4 °C. The results are expressed as mg MDA/Kg of product. An effect of the antioxidant, the packaging method and the time of storage was observed for this parameter (*p* < 0.05).

The initial value of the TBARS ranged from 0.20 to 0.66 (*p* < 0.05). This range of values at Day 0 can be related to the differences in the fat content among the samples.

Regarding the packaging method, the TBARS values of the samples packaged in MAP were higher than those packaged in OW conditions (*p* > 0.05), regardless of the antioxidant used or the day of refrigerated storage. These higher TBARS values are due to the higher oxygen content in the samples packaged in MAP (80%), which leads to an increased susceptibility of minced beef to lipid oxidation, with associated adverse effects on color [35].

With respect to the antioxidant added, in patties packaged in MAP conditions, the TBARS values of the patties with OE added (OE1-MA and OE2-MA) were lower than those obtained in patties with sulfite (S-MAP) or without additives (C-MAP) from Day 5 of treatment. Likewise, the higher dose of OE, the higher protection against lipid oxidation, was observed in MAP beef patties. The sulfites did not protect the beef patties from the rancidity since the TBARS values of the patties with (S-MAP) or without antioxidants (C-MAP) were similar. These results can be partially explained by the self-oxidation from the sulfite to a sulfate that can be accelerated by several factors, such as iron, a heme group of myoglobin and the partial pressure of oxygen [36]. Furthermore, Lizada and Yang [37] reported that sulfite could induce the direct oxidation of linoleic acid, one of the fatty acids present in beef, to low molecular weight compounds. This lipid oxidation needs oxygen and occurs together with the oxidation of sulfite to sulfate, which explains why the TBARS values in patties with sulfite in MAP (S-MAP) were so high.

The presence of OE also protects the patties from lipid oxidation in the samples packed in overwrap conditions (*p* < 0.05) through the storage time. The OE can slow TBARS formation due to its antioxidant activity as a consequence of its phenolic compounds, which act as free radical scavengers. The efficacy of the OE to delay the lipid oxidation in foal patties refrigerated for 6 days has been showed in a previous study [38]. Moreover, the treatment with sulfite (S-OW) had similar TBARS values to the treatments with OE throughout the refrigerated storage. This antioxidant activity of sulfites is due to its ability to break down hydroperoxides without producing radicals [39]. Likewise, sulfites can scavenge reactive oxygen species (ROS), ending the radical termination processes. In addition to this, sulfites might reduce other pro-oxidants, such as H_2_O_2_, and avoid the accumulation of protein radicals that could act as lipid pro-oxidants [23]. An interaction of packaging and antioxidant was found for this parameter during all storage times (*p* ≤ 0.05), except for Day 0 (*p* = 0.495).

The limit of detection for the rancidity for beef acceptability to consumers has been set at 2 mg MDA/kg meat [40]. Taking this value into account, this level of rancidity was reached by the control patties packaged in MAP conditions (C-MAP) at Day 2 (2.17 mg MDA/kg) and the control patties packaged in OW conditions (C-OW) at Day 5 (2.33 mg MDA/kg). Patties with sulfites and packaged in OW conditions (S-OW) had TBARS values lower than 2 mg MDA/kg during the 15 days of refrigerated storage. However, the combination of the addition of sulfites with MAP packing led to TBARS values similar to those obtained from patties without antioxidant added (C-MAP). It should be noted that the OE delayed the lipid oxidation in the MAP and OW conditions, and the higher the dose used, the higher the antioxidant activity. The OE-containing samples showed mean TBARS values lower than 2 mg MDA/kg during the 15 days of refrigerated storage, except the OE1-MAP at Day 15. Therefore, the combination of 250 mg/kg OE with 25% polyphenols and 15% hydroxytyrosol, and MAP packaging, would be effective against lipid oxidation up to 15 days.

### 3.6. Volatile Organic Compounds

Fatty acids, amino acids, peptides, lipids and proteins are important contributors to meat flavor through hydrolysis, oxidation of lipids proteolysis and Maillard reactions [41]. In this sense, it is well-known that lipid oxidation involves the formation of free radicals and hydroperoxides whose decomposition can result in the formation of volatile compounds (aldehydes, alcohols, ketones, etc.). Many of these volatile compounds have an unpleasant flavor and are responsible for the rancid odors present in meat products during storage [21]; thus, these compounds are reliable parameters for monitoring the shelf-life of meat products. Among these VOCs, aldehydes, and mainly hexanal, are extensively recognized as markers of lipid oxidation in meat products. Other VOCs that are also related to the formation of rancid odors are alcohols, 2,3-octanedione and 2-pentylfuran [22]. Table 8 shows the following VOCs studied: hexanal, 2-pentyl-furan, 1-pentanol, 2,3-octanedione, hexanol, nonanal and 1-octen-2-ol.

According to the TBARS results, the OE delayed the formation of all the volatile compounds studied, since in general the patties had significantly lower amounts of all the VOCs evaluated at Days 5, 8 and 12 than the control samples in both methods of packaging studied. An effect of the dose added was also found, so the higher the amount of OE added the higher the antioxidant effect. The addition of 250 mg of OE/kg of product inhibited the formation of 2-pentylfural and nonanal over the storage time studied. An effect of the type of packaging was also found, mainly after Day 5 of treatment, except for the compounds 1-octen-2-ol and 2,3-octanedione. The OW control samples showed higher concentrations of hexanal, 2,3-octanedione and 1-octen-2-ol than those of the MAP control patties, but lower concentrations than the other four volatile compounds studied. However, in all the cases, the antioxidant effect of the OE was higher in the samples packaged in OW conditions than in MAP with a high oxygen content. A combination effect of the packaging and antioxidant employed was also found (*p* ≤ 0.05), with the exception of nonanal.

These results also showed higher effectiveness of the OE than sulfites, highlighting the high potential of this OE as an antioxidant in beef patties stored in OW or MAP. It was expected that the sulfites show significantly lower levels of hexanal, as well as the rest of the VOCs studied, than the control samples due to its antioxidant activity [39]; however in the present study, sulfites only delayed the formation of 2-pentylfuran, 2,3 octanedione and 1-pentanol in both kinds of packaging methods studied, and hexanol and 1-octen-2-ol in the OW samples. These results agree with those previously discussed for the TBARS values.

Regarding to the evolution of the VOC analyzed, the general trend was an increase during the first 8–12 days of storage, after which the concentration decreased. In those cases where an effect of storage time was not observed, the level of the volatile compound studied remained constant. This finding agrees with the results found by Tateo et al. [42], who reported that the amount of many volatile compounds of foal meat packaged under vacuum conditions decreased after 9 days of storage. This decrease during the storage time can be related to the fact that hexanal and the other VOCs are formed in the early stages of the oxidation process and undergo further reactions that can be responsible of their decrease [43]. Interactions between proteins and lipid oxidation products can take place from Shiff bases via condensation reactions [44]. The antioxidant effect of an olive extract on volatile organic compounds formed during lipid oxidation of beef meat can be due to the polyphenols present in the extract; however, this is the first study of such an approach in the literature. It should be taken into account that the most important polyphenol present in olive oil and its wastes is hydroxytyrosol, which is a phenolic compound with a high antioxidant activity [45]. The antioxidant mechanisms of polyphenols are usually ascribed to their capacity to donate electrons to free radicals formed during lipid oxidation and to their capacity to stabilize their structure by resonance delocalization of an electron within their aromatic ring [4]. Other products with a high phenolic content, such as wine pomace or herbs and spices, have also showed the ability to protect meat products from lipid oxidation [7,46].

## 4. Conclusions

The addition an OE delayed the loss of redness, and the color protective effect of the OE was greater in patties stored in MAP. The addition of an OE protected the patties from lipid oxidation in the samples packed in both OW and MAP. According to our results, 250 mg/Kg of OE would be effective to protect beef patties against lipid oxidation for up to 15 days. The OE has inhibited the formation of all the volatile compounds studied with both kind of packaging, and its effectiveness was higher than that of chemical sulfites. Furthermore, the result obtained raises a question with respect to the roles associated with these VOCs, mainly hexanal, as markers of lipid oxidation in meat products. Moreover, the antimicrobial activity of the OE, at the doses studied, was insufficient to extend the shelf-life of the patties.

Therefore, the use of a natural antioxidant, such as an OE, can boost a transition towards healthier meat products, with a higher content in antioxidant components while free of harmful chemicals, such as sulfite. Considering the growing demand for healthier food products, the meat products treated with natural antioxidants might have broad acceptance among consumers. Additionally, olive producers might benefit from the added value of their olive fruits as a source of natural antioxidants for the food industry.

Nonetheless further research would be necessary to evaluate the addition of this extract at higher doses or in combination with hurdle technologies, together with the impact on the consumers’ acceptability of these new meat products to be used as a natural antioxidant in beef patties.

## Figures and Tables

**Table 1:** Proximate composition of the beef patties at Day 0 (mean values ± standard deviations).

Parameter (%)	Formulations				*p*-Value
	C	S	OE1	OE2	
Moisture	70.58 ± 2.30	70.97 ± 1.05	71.15 ± 1.46	71.04 ± 1.05	0.983
Fat	7.88 ± 1.70	7.70 ± 1.29	7.71 ± 0.91	7.70 ± 1.40	0.999
Protein	19.12 ± 1.46	19.70 ± 0.37	19.45 ± 0.59	19.64 ± 0.59	0.903

C (control), without antioxidant; S, 300 mg potassium metabisulfite/kg; OE1, 150 mg OE/kg; OE2, 250 mg OE/kg. OE: olive extract.

**Table 2:** Evolution of a_w_ and pH (mean values ± standard deviations) of beef patties with different formulations ^α^ packed under different conditions ^β^ during 15 days of storage at 4 °C.

Parameter	Packaging	Antioxidant	Days of Storage						
			0	2	5	8	12	15	Storage
**pH**	OW	C	5.78 ^aw^ ± 0.17	5.76 ^aw^ ± 0.06	5.78 ^aw^ ± 0.10	5.65 ^abw^ ± 0.02	5.44 ^bx^ ± 0.08	5.57 ^abx^ ± 0.00	0.001
		S	5.72 ^aw^ ± 0.10	5.77 ^aw^ ± 0.05	5.81 ^aw^ ± 0.13	5.78 ^abw^ ± 0.03	5.71 ^aw^ ± 0.04	5.88 ^aw^ ± 0.01	0.204
		OE1	5.71 ^abw^ ±0.06	5.79 ^aw^ ± 0.02	5.80 ^aw^ ± 0.10	5.59 ^bcw^ ± 0.02	5.48 ^cx^ ± 0.07	5.60 ^bcy^ ± 0.01	<0.001
		OE2	5.72 ^abw^ ± 0.10	5.75 ^abw^ ± 0.09	5.79 ^aw^ ± 0.10	5.61 ^abw^ ± 0.04	5.52 ^bx^ ± 0.14	5.59 ^aby^ ± 0.01	0.009
	MAP	C	5.75 ^abw^ ± 0.11	5.84 ^aw^ ± 0.07	5.80 ^aw^ ± 0.10	5.58 ^bcx^ ± 0.02	5.54 ^cx^ ± 0.10	5.67 ^abcy^ ± 0.01	<0.001
		S	5.74 ^aw^ ± 0.07	5.81 ^aw^ ± 0.02	5.78 ^aw^ ± 0.08	5.71 ^aw^ ± 0.06	5.71 ^aw^ ± 0.03	5.80 ^aw^ ± 0.01	0.066
		OE1	5.75 ^abw^ ± 0.10	5.80 ^aw^ ± 0.03	5.79 ^aw^ ± 0.07	5.63 ^bcwx^ ± 0.05	5.55 ^cx^ ± 0.06	5.59 ^bcz^ ± 0.01	<0.001
		OE2	5.72 ^abw^ ± 0.07	5.81 ^aw^ ± 0.01	5.79 ^aw^ ± 0.08	5.60 ^bcx^ ± 0.03	5.54 ^cx^ ± 0.06	5.59 ^cz^ ± 0.01	<0.001
		Packaging	0.933	0.011	0.885	0.055	0.120	0.824	
	Antioxidant		0.865	0.831	0.977	<0.001	<0.001	<0.001	
	Packaging × Antioxidant		0.933	0.674	0.964	0.030	0.593	<0.001	
**a_w_**	OW	C	0.980 ^aw^ ± 0.003	0.984 ^aw^ ± 0.003	0.984 ^aw^ ± 0.001	0.982 ^aw^ ± 0.002	0.982 ^aw^ ± 0.002	0.981 ^aw^ ± 0.002	0.233
		S	0.981 ^aw^ ± 0.002	0.981 ^aw^ ± 0.002	0.982 ^aw^ ± 0.001	0.984 ^aw^ ± 0.001	0.983 ^aw^ ± 0.002	0.983 ^aw^ ± 0.002	0.193
		OE1	0.979 ^bw^ ± 0.003	0.983 ^abw^ ± 0.003	0.983 ^abw^ ± 0.001	0.983 ^abw^ ± 0.002	0.983 ^abw^ ± 0.002	0.986 ^aw^ ± 0.001	0.049
		OE2	0.980 ^aw^ ± 0.003	0.984 ^aw^ ± 0.003	0.985 ^aw^ ± 0.002	0.981 ^aw^ ± 0.002	0.983 ^aw^ ± 0.002	0.982 ^aw^ ± 0.000	0.089
	MAP	C	0.978 ^bw^ ± 0.002	0.983 ^aw^ ± 0.002	0.984 ^aw^ ± 0.001	0.984 ^aw^ ± 0.002	0.982 ^abw^ ± 0.002	0.984 ^aw^ ± 0.000	0.008
		S	0.982 ^aw^ ± 0.003	0.983 ^aw^ ± 0.001	0.983 ^aw^ ± 0.001	0.982 ^aw^ ± 0.003	0.982 ^aw^ ± 0.002	0.984 ^aw^ ± 0.003	0.933
		OE1	0.977 ^bw^ ± 0.001	0.983 ^abw^ ± 0.001	0.984 ^aw^ ± 0.002	0.982 ^abw^ ± 0.003	0.982 ^abw^ ± 0.005	0.986 ^aw^ ± 0.001	0.012
		OE2	0.980 ^aw^ ± 0.002	0.983 ^aw^ ± 0.003	0.983 ^aw^ ± 0.002	0.981 ^aw^ ± 0.003	0.982 ^aw^ ± 0.006	0.983 ^aw^ ± 0.003	0.569
		Packaging	0.460	0.643	0.692	0.934	0.506	0.267	
		Antioxidant	0.081	0.593	0.252	0.235	0.967	0.096	
	Packaging × Antioxidant		0.548	0.705	0.045	0.428	0.995	0.677	

^a,b,c,d^: Means within a row with different letters are significantly different, *p* < 0.05 (effect of storage time). ^w,x,y,z^: means within a column and type of packaging (OW or MAP) with different letters are significantly different, *p* < 0.05 (effect of antioxidant). ^α^ Formulations: C (control), without antioxidant; S, 300 mg potassium metabisulfite/kg; OE1, 150 mg OE/kg; OE2, 250 mg OE/kg. ^β^ Packaging conditions: MAP, modified atmosphere with a high-oxygen atmosphere (80% O2/20% CO2); OW: overwrap. OE: olive extract. The Storage, Antioxidant, Packaging and Packaging × Antioxidant words represent *p*-values.

**Table 3:** Evolution of the color parameters (mean values ± standard deviations) of beef patties with different formulations ^α^ packed under different conditions ^β^ during storage at 4 °C.

Parameter	Packaging	Antioxidant	Days of Storage						
			0	2	5	8	12	15	Storage
L*	OW	C	29.95 ^aw^ ± 2.91	28.13 ^aw^ ± 2.26	29.83 ^aw^ ± 2.27	29.65 ^aw^ ± 2.58	28.56 ^aw^ ± 2.16	27.02 ^aw^ ± 1.40	0.144
		S	29.78 ^aw^ ± 2.12	28.37 ^aw^ ± 1.72	27.96 ^aw^ ± 3.56	28.03 ^aw^ ± 2.02	28.05 ^aw^ ± 2.95	27.04 ^aw^ ± 1.56	0.409
		OE1	29.90 ^aw^ ± 1.82	28.64 ^aw^ ± 1.27	29.06 ^aw^ ± 2.06	28.30 ^aw^ ± 3.04	29.99 ^aw^ ± 1.14	31.14 ^aw^ ± 2.33	0.098
		OE2	29.21 ^aw^ ± 2.67	28.42 ^aw^ ± 1.68	29.49 ^aw^ ± 1.90	29.16 ^aw^ ± 2.27	29.20 ^aw^ ± 1.10	30.46 ^aw^ ± 1.64	0.579
	MAP	C	29.32 ^bw^ ± 2.95	28.11 ^bw^ ± 2.13	28.24 ^bw^ ± 1.41	31.06 ^abw^ ± 2.71	32.85 ^aw^ ± 0.84	32.68 ^aw^ ± 1.19	<0.001
		S	29.28 ^aw^ ± 2.25	30.19 ^aw^ ± 1.59	29.15 ^aw^ ± 2.27	28.38 ^aw^ ± 3.41	30.69 ^aw^ ± 2.18	30.18 ^awx^ ± 1.89	0.309
		OE1	29.64 ^aw^ ± 2.75	29.83 ^aw^ ± 1.86	29.55 ^aw^ ± 3.67	29.55 ^aw^ ± 1.82	30.79 ^aw^ ± 1.47	29.41 ^ax^ ± 1.84	0.835
		OE2	30.64 ^aw^ ± 2.17	29.75 ^aw^ ± 2.00	30.54 ^aw^ 3.08	31.24 ^aw^ ± 1.86	31.63 ^aw^ ± 2.15	28.40 ^ax^ ± 1.51	0.123
		Packaging	0.984	0.010	0.630	0.027	<0.001	0.027	
		Antioxidant	0.962	0.160	0.371	0.053	0.132	0.049	
	Packaging × Antioxidant		0.530	0.442	0.322	0.755	0.041	<0.001	
a*	OW	C	22.26 ^aw^ ± 2.18	9.37 ^by^ ± 2.05	8.19 ^bx^ ± 0.95	8.63 ^bw^ ± 2.84	7.98 ^bwx^ ± 1.46	7.78 ^bw^ ± 1.21	<0.001
		S	21.77 ^aw^ ± 1.64	16.81 ^bw^ ± 2.90	16.33 ^bw^ ± 3.66	7.89 ^cx^ ± 1.09	7.61 ^cx^ ± 0.82	7.69 ^cw^ ± 0.78	<0.001
		OE1	21.74 ^aw^ ± 1.93	12.70 ^bx^ ± 2.61	7.98 ^cx^ ± 1.24	8.34 ^cwx^ ± 0.73	9.02 ^cw^ ± 1.05	7.96 ^cw^ ± 1.12	<0.001
		OE2	18.85 ^aw^ ± 2.67	12.92 ^bx^ ± 2.71	8.03 ^cx^ ± 1.64	7.78 ^cx^ ± 1.46	9.19 ^cw^ ± 1.08	6.92 ^cw^ ± 0.65	<0.001
	MAP	C	18.03 ^aw^ ± 4.03	15.18 ^ax^ ± 2.08	11.78 ^bx^ ± 1.60	7.12 ^cx^ ± 1.05	5.82 ^cx^ ± 1.21	6.01 ^cw^ ± 0.58	<0.001
		S	20.79aw ± 3.13	18.37 ^aw^ ± 1.61	17.54 ^aw^ ± 3.52	12.70 ^bw^ ± 2.25	5.80 ^cx^ ± 0.62	6.00 ^cw^ ± 0.92	<0.001
		OE1	20.50 ^aw^ ± 3.04	17.19 ^bwx^ ± 1.48	15.06 ^bw^ ± 1.67	11.48 ^cw^ ± 1.57	6.33 ^dwx^ ± 0.71	6.10 ^dw^ ± 0.71	<0.001
		OE2	19.74 ^aw^ ± 2.08	17.23 ^bwx^ ± 2.38	14.97 ^bw^ ± 1.38	12.43 ^cw^ ± 1.66	7.17 ^dw^ ± 0.54	6.69 ^dw^ ± 0.72	<0.001
		Packaging	0.024	<0.001	<0.001	<0.001	<0.001	<0.001	
		Antioxidant	0.077	<0.001	<0.001	<0.001	<0.001	0.942	
	Packaging × Antioxidant		0.032	0.034	<0.001	<0.001	0.530	0.131	
b*	OW	C	20.03 ^aw^ ± 2.05	11.68 ^bcx^ ± 1.76	12.90 ^bwx^ ± 1.87	12.70bw ± 1.61	11.69bcw ± 1.71	9.92 ^cw^ ± 0.82	<0.001
		S	19.28 ^aw^ ± 2.12	16.51 ^abw^ ± 2.27	15.77 ^bw^ ± 3.70	11.69 ^cw^ ± 1.76	11.13cw ± 1.63	10.17 ^cw^ ± 1.29	<0.001
		OE1	19.36 ^aw^ ± 2.29	14.72 ^bw^ ± 2.03	12.90 ^bcwx^ ± 2.08	11.88 ^cw^ ± 1.82	12.01cw ± 1.32	11.04 ^cw^ ± 1.07	<0.001
		OE2	17.61 ^aw^ ± 2.35	14.26 ^bw^ ± 2.15	12.58 ^bcx^ ± 2.43	11.56 ^bcdw^ ± 1.40	11.04cdw ± 1.67	9.45 ^dw^ ± 1.72	<0.001
	MAP	C	16.75 ^aw^ ± 1.93	16.04 ^abx^ ± 0.77	15.01 ^abx^ ± 1.55	13.91 ^bw^ ± 1.48	14.22bw ± 1.42	15.50 ^abw^ ± 1.62	0.001
		S	17.76 ^aw^ ± 2.90	16.93 ^abwx^ ± 1.54	17.21 ^abw^ ± 3.09	14.52 ^bcw^ ± 1.62	12.53 ^cw^ ± 0.98	13.75 ^cw^ ± 1.88	<0.001
		OE1	18.72 ^aw^ ± 3.09	17.17 ^aw^ ± 1.84	16.30 ^awx^ ± 2.05	13.90 ^bcw^ ± 1.84	12.71 ^cw^ ± 1.12	13.12 ^cw^ ± 1.36	<0.001
		OE2	18.00 ^aw^ ± 2.56	16.36 ^abx^ ± 2.67	15.84 ^abcx^ ± 1.88	14.54 ^bcdw^ ± 2.37	13.18 ^cdw^ ± 1.59	12.45 ^dw^ ± 1.28	<0.001
		Packaging	0.024	<0.001	<0.001	<0.001	<0.001	<0.001	
		Antioxidant	0.462	<0.001	0.006	0.901	0.096	0.067	
	Packaging × Antioxidant		0.116	0.022	0.524	0.364	0.206	0.060	

^a,b,c,d^: Means within a row with different letters are significantly different, *p* < 0.05 (effect of storage time). ^w,x,y,z^: means within a column and type of packaging, (OW or MAP), with different letters are significantly different, *p* < 0.05 (effect of antioxidant). ^α^ Formulations: C (control), without antioxidant; S, 300 mg potassium metabisulfite/kg; OE1, 150 mg OE/kg; OE2, 250 mg OE/kg. ^β^ Packaging conditions: MAP, modified atmosphere with a high-oxygen atmosphere (80% O2/20% CO2); OW: overwrap. OE: olive extract. The Storage, Antioxidant, Packaging and Packaging × Antioxidant words represent *p*-values.

**Table 4:** The total color difference parameter (ΔE*) between the control patties and the patties with antioxidants ^α^ packed under different conditions ^β^ during storage at 4 °C.

Parameter	Packaging	Antioxidant	Days of Storage					
			0	2	5	8	12	15
ΔE*	OW	C						
		S	1.15 ^x^ ± 0.06	9.02 ± 1.69	9.45 ^w^ ± 0.53	3.41 ± 0.01	2.92 ± 0.79	3.83 ± 0.47
		OE1	1.56 ^wx^ ± 0.23	4.56 ± 0.56	0.94 ^x^ ± 0.65	2.52 ± 0.80	2.18 ± 0.12	3.98 ± 0.39
		OE2	4.28 ^w^ ± 0.79	4.55 ± 1.1	1.63 ^x^ ± 0.61	1.86 ± 0.15	2.43 ± 0.86	7.24 ± 0.62
	MAP	C						
		S	3.02 ± 0.16	3.74 ± 0.17	5.49 ± 0.93	7.05 ± 0.30	2.14 ± 1.04	1.68 ± 0.54
		OE1	4.25 ± 2.80	3.12 ± 0.36	4.12 ± 1.70	5.11 ± 1.93	2.67 ± 1.06	2.43 ± 0.76
		OE2	2.79 ± 0.97	2.91 ± 1.54	4.08 ± 1.97	5.53 ± 0.36	2.03 ± 1.13	3.64 ± 0.62
		Packaging	0.818	0.082	0.972	0.033	0.823	0.141
		Antioxidant	0.365	0.191	0.022	0.464	0.875	0.080
Packaging × Antioxidant			0.198	0.092	0.031	0.503	0.613	0.107

^w,x^: Means within a column and type of packaging (OW or MAP) with different letters are significantly different, *p* < 0.05 (effect of antioxidant). ^α^ Formulations: C (control), without antioxidant; S, 300 mg potassium metabisulfite/kg; OE1, 150 mg OE/kg; OE2, 250 mg OE/kg. ^β^ Packaging conditions: MAP, modified atmosphere with a high-oxygen atmosphere (80% O2/20% CO2); OW: overwrap. OE: olive extract. The Storage, Antioxidant, Packaging and Packaging × Antioxidant words represent *p*-values.

**Table 5:** The total color difference parameter (ΔE*) between patties at Day 0 and the rest of the days ^α^ packed under different conditions ^β^ during storage at 4 °C.

Parameter	Packaging	Antioxidant		Days of Storage					
			0	2	5	8	12	15	Storage
ΔE*	OW	C		15.51 ± 0.34	15.96 ± 0.53	15.49 ± 3.09	15.13 ± 0.49	15.18 ± 0.29	0.975
		S		6.17 ^b^ ± 2.72	6.74 ^b^ ± 2.93	15.94 ^a^ ± 0.50	16.46 ^a^ ± 0.75	15.70 ^a^ ± 0.40	0.005
		OE1		10.5 ^b^ ± 1.49	15.25 ^a^ ± 1.29	15.53 ^a^ ± 0.89	14.72 ^a^ ± 1.19	14.91 ^a^ ± 0.58	0.024
		OE2		7.04 ^b^ ± 0.21	12.01 ^a^ ± 0.46	12.61 ^a^ ± 3.08	11.80 ^a^ ± 0.78	13.35 ^a^ ± 0.52	0.040
	MAP	C		3.39 ^c^ ± 1.47	6.61 ^b^ ± 1.70	11.61 ^a^ ± 1.39	12.83 ^a^ ± 0.18	11.04 ^a^ ± 0.01	0.002
		S		3.22 ^c^ ± 3.17	4.31 ^b^ ± 2.65	9.22 ^a^ ± 0.84	13.55 ^a^ ± 0.14	13.19 ^a^ ± 0.097	0.007
		OE1		3.94 ^b^ ± 3.39 ^b^	6.02 ^ab^ ± 2.60	10.30 ^a^ ± 2.01	15.53 ^a^ ± 3.29	13.96 ^a^ ± 1.75	0.029
		OE2		9.58 ± 8.19	11.80 ± 7.80	14.64 ± 6.07	20.00 ± 6.29	20.38 ± 6.35	0.505

^a,b,c^: Means within a row with different letters are significantly different, *p* < 0.05 (effect of storage time). ^α^ Formulations: C (control), without antioxidant; S, 300 mg potassium metabisulfite/kg; OE1, 150 mg OE/kg; OE2, 250 mg OE/kg. ^β^ Packaging conditions: MAP, modified atmosphere with a high-oxygen atmosphere (80% O2/20% CO2); OW: overwrap. OE: olive extract. The Storage, Antioxidant, Packaging and Packaging × Antioxidant words represent *p*-values.

**Table 6:** Evolution of the total viable count (mean values ± standard deviations) of beef patties with different formulations ^α^ packed under different conditions ^β^ during storage at 4 °C.

Parameter	Packaging	Antioxidant		Days of Storage					
			0	2	5	8	12	15	Storage
Log cfu/g	OW	C	4.35 ^cw^ ± 0.40	4.84 ^bcwx^ ± 0.12	5.69 ^bw^ ± 1.22	7.89 ^aw^ ± 0.10	7.69 ^aw^ ± 0.06	7.96 ^aw^ ± 0.03	<0.001
		S	4.31 ^dw^ ± 0.16	4.52 ^dx^ ± 0.32	5.96 ^bcw^ ± 0.53	6.71 ^ay^ ± 0.20	5.90 ^cy^ ± 0.88	6.64 ^abx^ ± 0.04	<0.001
		OE1	4.38 ^d1w^ ± 0.15	4.93 ^cw^ ± 0.08	6.93 ^bw^ ± 0.37	7.88 ^aw^ ± 0.23	7.63 ^abw^ ± 0.19	7.85 ^aw^ ± 0.05	<0.001
		OE2	4.34 ^e1w^ ± 0.24	5.10 ^dw^ ± 0.18	6.09 ^cw^ ± 1.04	7.45 ^aby^ ± 0.23	6.85 ^bx^ ± 0.20	7.82 ^aw^ ± 0.19	<0.001
	MAP	C	4.35 ^d1w^ ± 0.40	4.51 ^dw^ ± 0.25	5.76 ^cw^ ± 0.26	7.65 ^bw^ ± 0.40	7.12 ^bw^ ± 0.21	8.42 ^aw^ ± 0.03	<0.001
		S	4.31 ^c1w^ ± 0.16	3.72 ^cx^ ± 0.57	4.57 ^cw^ ± 0.99	5.37 ^bcy^ ± 1.59	6.67 ^abx^ ± 0.84	7.92 ^ax^ ± 0.01	<0.001
		OE1	4.38 ^c1w^ ± 0.15	4.69 ^cw^ ± 0.26	6.02 ^bw^ ± 1.04	7.74 ^aw^ ± 0.31	7.28 ^aw^ ± 0.28	8.38 ^aw^ ± 0.03	<0.001
		OE2	4.34 ^ew^ ± 0.24	4.98 ^dew^ ± 0.13	5.62 ^cdw^ ± 1.62	7.74 ^abw^ ± 0.30	6.80 ^bcwx^ ± 0.80	8.23 ^w^ ± 0.03	<0.001
		Packaging	0.997	<0.001	0.021	0.080	0.837	<0.001	
		Antioxidant	0.880	<0.001	0.059	<0.001	<0.001	<0.001	
	Packaging × Antioxidant		0.989	0.078	0.310	0.015	0.014	<0.001	

^a,b,c,d^: Means within a row with different letters are significantly different, *p* < 0.05 (effect of storage time). ^w,x,y,z^: means within a column and type of packaging (OW or MAP) with different letters are significantly different, *p* < 0.05 (effect of antioxidant). ^α^ Formulations: C (control), without antioxidant; S, 300 mg potassium metabisulfite/kg; OE1, 150 mg OE/kg; OE2, 250 mg OE/kg. ^β^ Packaging conditions: MAP, modified atmosphere with a high-oxygen atmosphere (80% O2/20% CO2); OW: overwrap. OE: olive extract. The Storage, Antioxidant, Packaging and Packaging × Antioxidant words represent *p*-values.

**Table 7:** Evolution of the TBARS (mg MDA/Kg) values (mean values ± standard deviations) of beef patties with different formulations^α^ packed under different conditions^β^ during storage at 4 °C.

Parameter	Packaging	Antioxidant	Days of Storage						
			0	2	5	8	12	15	Storage
**TBARS**	OW	C	0.66 ^cw^ ± 0.03	1.26 ^bcw^ ± 0.05	2.33 ^aw^ ± 0.43	2.23 ^aw^ ± 0.55	1.84 ^abw^ ± 0.08	1.12 ^bcw^ ± 0.05	<0.001
		S	0.20 ^ay^ ± 0.03	0.31 ^ay^ ± 0.01	0.43 ^ay^ ± 0.10	0.60 ^ay^ ± 0.14	0.95 ^ax^ ± 0.76	0.38 ^ay^ ± 0.03	0.272
		OE1	0.45 ^ax^ ± 0.03	0.79 ^ax^ ± 0.15	1.08 ^ax^ ± 0.23	0.93 ^ay^ ± 0.40	1.05 ^awx^ ± 0.07	0.81 ^ax^ ± 0.14	0.103
		OE2	0.33 ^bx^ ± 0.02	0.64 ^ax^ ± 0.05	0.69 ^axy^ ± 0.05	0.64 ^ay^ ± 0.08	0.71 ^ax^ ± 0.09	0.65 ^ax^ ± 0.03	0.001
	MAP	C	0.62 ^cw^ ± 0.07	2.17 ^bw^ ± 0.21	3.04 ^abw^ ± 0.51	2.94 ^abw^ ± 0.56	3.42 ^aw^ ± 0.28	4.01 ^aw^ ± 0.34	<0.001
		S	0.23 ^cy^ ± 0.00	0.93 ^bcxy^ ± 0.18	2.89 ^aw^ ± 0.33	2.80 ^abw^ ± 0.92	2.57 ^abwx^ ± 0.88	4.41 ^aw^ ± 0.34	<0.001
		OE1	0.50 ^cwx^ ± 0.07	1.38 ^bx^ ± 0.11	1.67 ^bx^ ± 0.19	1.54 ^bx^ ± 0.20	1.93 ^abxy^ ± 0.33	2.45 ^ax^ ± 0.13	<0.001
		OE2	0.34 ^cxy^ ± 0.03	0.53 ^bcy^ ± 0.20	1.10 ^ax^ ± 0.11	0.95 ^abx^ ± 0.24	0.99 ^aby^ ± 0.20	1.19 ^ay^ ± 0.28	0.003
	Packaging		0.542	<0.001	<0.001	<0.001	<0.001	<0.001	
	Antioxidant		<0.001	<0.001	<0.001	<0.001	<0.001	<0.001	
	Packaging × Antioxidant		0.495	0.005	<0.001	0.002	0.019	<0.001	

^a,b,c,d^: Means within a row with different letters are significantly different, *p* < 0.05 (effect of storage time). ^w,x,y,z^: means within a column and type of packaging (OW or MAP) with different letters are significantly different, *p* < 0.05 (effect of antioxidant). ^α^ Formulations: C (control), without antioxidant; S, 300 mg potassium metabisulfite/kg; OE1, 150 mg OE/kg; OE2, 250 mg OE/kg. ^β^ Packaging conditions: MAP, modified atmosphere with a high-oxygen atmosphere (80% O2/20% CO2); OW: overwrap. OE: olive extract. The Storage, Antioxidant, Packaging and Packaging × Antioxidant words represent *p*-values.

**Table 8:** The volatile organic compounds (in area accounts × 10^−6^, arbitrary units) of beef patties with different formulations packed under different conditions during storage at 4 °C.

Compound	Pack.	Anti.	Days of Storage					
			0	5	8	12	15	Storage
**Hexanal**	OW	C	0.077 ^ax^ ± 0.015	0.543 ^bcw^ ± 0.012	0.522 ^bcx^ ± 0.080	0.451 ^bx^ ± 0.055	0.682 ^cy^ ± 0.047	<0.001
		S	N.D.	0.512 ^aw^ ± 0.280	0.562 ^ax^ ± 0.070	0.697 ^ax^ ± 0.156	0.458 ^ax^ ± 0.062	0.355
		OE1	N.D.	0.041 ^aw^ ± 0.001	0.048 ^aw^ ± 0.006	0.058 ^aw^ ± 0.014	0.041 ^aw^ ± 0.001	0.152
		OE2	N.D.	N.D.	0.015 ^aw^ ± 0.001	0.027 ^bw^ ± 0.003	0.049 ^cw^ ± 0.002	<0.001
	MAP	C	N.D.	0.535 ^bx^ ± 0.155	0.319 ^ax^ ± 0.033	0.236 ^ay^ ± 0.015	0.182 ^ax^ ± 0.033	0.006
		S	N.D.	0.500 ^abx^ ± 0.114	0.706 ^by^ ± 0.110	0.310 ^az^ ± 0.018	0.267 ^ay^ ± 0.012	0.005
		OE1	N.D.	0.188 ^awx^ ± 0.097	0.128 ^awx^ ± 0.003	0.162 ^ax^ ± 0.022	0.170 ^ax^ ± 0.011	0.579
		OE2	N.D.	0.038 ^aw^ ± 0.002	0.026 ^aw^ ± 0.011	0.043 ^aw^ ± 0.006	0.037 ^aw^ ± 0.003	0.103
	Packaging		_	0.605	0.788	<0.001	<0.001	
	Antioxidant		_	0.004	<0.001	<0.001	<0.001	
Packaging × Antioxidant			_	0.650	0.011	<0.001	<0.001	
**2-pentyl-furan**	OW	C	N.D.	0.192 ^bx^ ± 0.010	0.169 ^by^ ± 0.013	0.121 ^ax^ ± 0.014	0.095 ^aw^ ± 0.008	0.001
		S	N.D.	0.074 ^abcw^ ± 0.001	0.087 ^bx^ ± 0.007	0.072 ^aw^ ± 0.006	0.083 ^abw^ ± 0.003	0.028
		OE1	N.D.	0.030 ^w^ ± 0.001	N.D.	N.D.	N.D.	_
		OE2	N.D.	N.D.	N.D.	N.D.	N.D.	_
	MAP	C	N.D.	0.207 ^bx^ ± 0.004	0.136 ^ax^ ± 0.007	0.191 ^bx^ ± 0.019	0.138 ^aw^ ± 0.020	0.006
		S	N.D.	N.D.	0.104 ^bw^ ± 0.007	0.073 ^aw^ ± 0.001	0.134 ^cw^ ± 0.006	<0.001
		OE1	N.D.	0.106 ^acw^ ± 0.006	0.172 ^by^ ± 0.003	0.095 ^aw^ ± 0.021	0.118 ^aw^ ± 0.015	<0.001
		OE2	N.D.	N.D.	N.D.	N.D.	N.D.	_
	Packaging		_	0.059	<0.001	<0.001	<0.001	
	Antioxidant		_	<0.001	<0.001	<0.001	0.138	
Packaging × Antioxidant			_	_	<0.001	<0.001	0.625	
								
**1-Pentanol**	OW	C	0.047 ^aw^ ± 0.006	0.136 ^bcx^ ± 0.012	0.137 ^bcz^ ± 0.003	0.163 ^cy^ ± 0.018	0.095 ^by^ ± 0.013	<0.001
		S	N.D.	0.069 ^abwx^ ± 0.021	0.095 ^by^ ± 0.001	0.080 ^abx^ ± 0.005	0.060 ^ax^ ± 0.008	0.038
		OE1	0.031 ^aw^ ± 0.001	0.047 ^aw^ ± 0.022	0.053 ^ax^ ± 0.006	0.043 ^aw^ ± 0.006	0.032 ^aw^ ± 0.001	0.143
		OE2	N.D.	0.041 ^aw^ ± 0.016	0.022 ^aw^ ± 0.001	0.026 ^aw^ ± 0.006	0.035 ^aw^ ± 0.003	0.133
	MAP	C	N.D.	0.165 ^ax^ ± 0.003	0.196 ^ax^ ± 0.028	0.159 ^ay^ ± 0.018	0.156 ^awx^ ± 0.040	0.475
		S	N.D.	0.150 ^ax^ ± 0.001	0.208 ^bx^ ± 0.010	0.192 ^bz^ ± 0.003	0.159 ^ax^ ± 0.015	0.002
		OE1	N.D.	0.171 ^bx^ ± 0.014	0.167 ^abx^ ± 0.027	0.130 ^abx^ ± 0.003	0.126 ^awx^ ± 0.009	0.018
		OE2	N.D.	0.048 ^aw^ ± 0.001	0.042 ^aw^ ± 0.008	0.097 ^bw^ ± 0.001	0.091 ^bw^ ± 0.013	<0.001
	Packaging		_	<0.001	<0.001	<0.001	<0.001	
	Antioxidant		0.060	<0.001	<0.001	<0.001	<0.001	
Packaging × Antioxidant			_	0.001	0.004	<0.001	0.114	
**2,3-Octanedione**	OW	C	N.D.	0.154 ^ax^ ± 0.021	0.149 ^ax^ ± 0.002	0.159 ^ax^ ± 0.011	0.148 ^ax^ ± 0.003	0.058
		S	N.D.	0.106 ^cx^ ± 0.005	0.100 ^bcw^ ± 0.001	0.074 ^abw^ ± 0.014	0.057 ^aw^ ± 0.006	0.003
		OE1	N.D.	0.014 ^w^ ± 0.001	N.D.	N.D.	N.D.	_
		OE2	N.D.	N.D.	N.D.	N.D.	N.D.	_
	MAP	C	N.D.	0.046 ^aw^ ± 0.001	0.093 ^axy^ ± 0.004	0.076 ^ax^ ± 0.002	0.084 ^ax^ ± 0.026	0.081
		S	N.D.	0.066 ^aw^ ± 0.028	0.122 ^ay^ ± 0.030	0.090 ^ay^ ± 0.002	0.090 ^ax^ ± 0.002	0.122
		OE1	N.D.	0.030 ^aw^ ± 0.001	0.053 ^awx^ ± 0.002	0.086 ^by^ ± 0.005	0.089 ^bx^ ± 0.012	<0.001
		OE2	N.D.	0.024 ^abw^ ± 0.005	0.014 ^aw^ ± 0.002	0.019 ^aw^ ± 0.001	0.034 ^bw^ ± 0.005	0.003
	Packaging		_	0.001	0.099	0.001	0.084	
	Antioxidant		_	<0.001	0.001	<0.001	<0.001	
Packaging × Antioxidant			_	0.001	0.004	0.208	<0.001	
**Hexanol**	OW	C	0.016 ^aw^ ± 0.002	0.261 ^bx^ ± 0.009	0.283 ^by^ ± 0.006	0.243 ^by^ ± 0.017	0.228 ^bx^ ± 0.032	<0.001
		S	N.D.	0.039 ^aw^ ± 0.016	0.185 ^cx^ ± 0.016	0.048 ^abwx^ ± 0.001	0.067 ^bw^ ± 0.005	<0.001
		OE1	N.D.	0.036 ^aw^ ± 0.019	0.074 ^bw^ ± 0.015	0.061 ^abx^ ± 0.005	0.055 ^abw^ ± 0.002	0.051
		OE2	N.D.	0.010 ^aw^ ± 0.005	0.027 ^bw^ ± 0.004	0.027 ^bw^ ± 0.005	0.047 ^cw^ ± 0.003	<0.001
	MAP	C	N.D.	0.178 ^aw^ ± 0.015	0.785 ^cx^ ± 0.053	0.571 ^bcy^ ± 0.045	0.453 ^abxy^ ± 0.153	0.004
		S	N.D.	0.066 ^aw^ ± 0.048	0.513 ^bwx^ ± 0.146	0.623 ^by^ ± 0.054	0.610 ^by^ ± 0.044	0.002
		OE1	N.D.	0.384 ^ax^ ± 0.054	0.694 ^bx^ ± 0.198	0.368 ^ax^ ± 0.013	0.303 ^awx^ ± 0.030	0.012
		OE2	N.D.	0.025 ^aw^ ± 0.014	0.073 ^abw^ ± 0.002	0.120 ^bw^ ± 0.003	0.188 ^cw^ ± 0.028	<0.001
	Packaging		_	0.001	<0.001	<0.001	<0.001	
	Antioxidant		_	<0.001	<0.001	<0.001	<0.001	
Packaging × Antioxidant			_	<0.001	0.009	<0.001	0.001	
**Nonanal**	OW	C	N.D.	0.041 ^aw^ ± 0.001	0.030 ^aw^ ± 0.003	0.030 ^ax^ ± 0.001	0.032 ^aw^ ± 0.005	0.052
		S	N.D.	0.027 ^aw^ ± 0.006	0.039 ^bw^ ± 0.001	0.030 ^abx^ ± 0.004	0.030 ^abw^ ± 0.002	0.066
		OE1	N.D.	N.D.	N.D.	0.008 ^aw^ ± 0.002	N.D.	_
		OE2	N.D.	N.D.	N.D.	N.D.	N.D.	
	MAP	C	N.D.	0.034 ^bw^ ± 0.004	0.023 ^abwx^ ± 0.002	0.017 ^ax^ ± 0.002	0.018 ^awx^ ± 0.005	0.010
		S	N.D.	0.034 ^bw^ ± 0.003	0.033 ^bx^ ± 0.003	0.024 ^ay^ ± 0.001	0.023 ^ax^ ± 0.002	0.006
		OE1	N.D.	0.025 ^bw^ ± 0.005	0.017 ^aw^ ± 0.002	0.013 ^aw^ ± 0.001	0.110 ^aw^ ± 0.001	0.002
		OE2	N.D.	N.D.	N.D.	N.D.	N.D.	_
	Packaging		_	0.995	0.012	<0.001	0.001	
	Antioxidant		_	0.044	0.002	<0.001	0.018	
Packaging × Antioxidant			_	0.064	0.701	<0.001	0.138	
**1-Octen-2-ol**	OW	C	N.D.	0.471 ^cx^ ± 0.021	0.388 ^bcx^ ± 0.060	0.269 ^aby^ ± 0.032	0.196 ^ay^ ± 0.028	0.002
		S	N.D.	0.197 ^bw^ ± 0.008	0.287 ^cx^ ± 0.013	0.201 ^bx^ ± 0.020	0.141 ^ax^ ± 0.017	<0.001
		OE1	N.D.	0.140 ^bw^ ± 0.055	0.071 ^abw^ ± 0.001	0.053 ^aw^ ± 0.007	0.052 ^aw^ ± 0.010	0.025
		OE2	N.D.	0.100 ^bw^ ± 0.013	0.034 ^aw^ ± 0.002	0.028 ^aw^ ± 0.004	0.052 ^aw^ ± 0.009	<0.001
	MAP	C	N.D.	0.302 ^aw^ ± 0.118	0.206 ^ax^ ± 0.010	0.172 ^axy^ ± 0.025	0.144 ^awx^ ± 0.050	0.105
		S	N.D.	0.209 ^aw^ ± 0.028	0.296 ^by^ ± 0.003	0.204 ^ay^ ± 0.005	0.164 ^ax^ ± 0.016	<0.001
		OE1	N.D.	0.259 ^aw^ ± 0.148	0.137 ^ax^ ± 0.032	0.143 ^ax^ ± 0.012	0.137 ^awx^ ± 0.011	0.219
		OE2	N.D.	0.086 ^aw^ ± 0.037	0.049 ^aw^ ± 0.014	0.057 ^aw^ ± 0.020	0.071 ^aw^ ± 0.017	0.361
	Packaging		_	0.725	0.097	0.425	0.079	
	Antioxidant		_	0.003	<0.001	<0.001	<0.001	
Packaging × Antioxidant			_	0.116	0.001	<0.001	0.002	

^a,b,c,d^: Means within a row with different letters are significantly different, *p* < 0.05 (effect of storage time). ^w,x,y,z^: means within a column and type of packaging (OW or MAP) with different letters are significantly different *p* < 0.05 (effect of antioxidant). ^α^ Formulations: C (control), without antioxidant; S, 300 mg potassium metabisulfite/kg; OE1, 150 mg OE/kg; OE2, 250 mg OE/kg. ^β^ Packaging conditions: MAP, modified atmosphere with a high-oxygen atmosphere (80% O2/20% CO2); OW: overwrap. OE: olive extract. The Storage, Antioxidant, Packaging and Packaging × Antioxidant words represent *p*-values. N.D. = not detected.
